# Serum miR-95-3p is a diagnostic and prognostic marker for osteosarcoma

**DOI:** 10.1186/s40064-016-3640-0

**Published:** 2016-11-09

**Authors:** Junjie Niu, Yibao Sun, Qiaoge Guo, Dongju Niu, Bo Liu

**Affiliations:** 1 Department of Radiology, Zhengzhou Orthopedics Hospital, Zhengzhou, China; 2 Department of Minimally Invasive Spine Surgery, Zhengzhou Orthopedics Hospital, No. 58 Longhai Road, Zhengzhou, 450000 Henan People’s Republic of China; 3 Zhengzhou Key Laboratory of Bone and Joint Imaging, Zhengzhou, China; 4 Department of Radiology, Zhengzhou Second Hospital, Zhengzhou, China; 5 Department of Function, Zhengzhou Orthopedics Hospital, Zhengzhou, China

**Keywords:** Osteosarcoma, miR-95-3p, Serum, Diagnosis, Prognosis

## Abstract

It has been demonstrated that microRNAs (miRNAs or miRs) can act as prognostic and diagnostic markers, and potential therapeutic targets. miR-95-3p has been reported to be downregulated in osteosarcoma tissues, but its potential as a serum biomarker has not been assessed in human osteosarcoma. The purpose of the present study was to examine the expression levels of miR-95-3p in serum of patients with osteosarcoma and to investigate the diagnostic and prognostic value of miR-95-3p. The serum levels of miR-95-3p in osteosarcoma patients were detected by a real-time quantitative reverse transcription-polymerase chain reaction assay. Associations between miR-95-3p expression and various clinicopathological characteristics were analyzed using Chi square test. Differences in patient survival were determined using the Kaplan–Meier method and a log-rank test. A Cox proportional hazards regression analysis was used for multivariate analyses of prognostic values. Compared to healthy controls, the expression levels of miR-95-3p in serum of osteosarcoma patients were significantly decreased (*P* < 0.0001). Low miR-95-3p expression had significant association with clinical stage (*P* < 0.001) and metastasis (*P* < 0.001). The Kaplan–Meier curve showed that patients with high miR-95-3p expression survived significantly longer than patients with low miR-95-3p expression (*P* = 0.017). Multivariate analysis demonstrated that miR-95-3p expression level (*P* = 0.014) was an independent prognostic biomarker for overall survival. Our findings suggested that down-expression of serum miR-95-3p might be associated with poor prognosis of osteosarcoma patients, suggesting that decreased expression of serum miR-95-3p may serve as a valuable diagnostic/prognostic marker for osteosarcoma patients.

## Background

Osteosarcomas are aggressive neoplasms of the bone that occurs predominantly in adolescents and young adults. Due to the low incidence of osteosarcomas and strong heterogeneous nature of osteosarcoma, the molecular pathogenesis of osteosarcoma is not well understood compared to other cancer types. The 5-year overall and disease-free survival rates for osteosarcoma patients are approximately 50–60%, and about 40% of patients died from lung metastases (Bruland et al. 2010). In recent years, neoadjuvant chemotherapy with cisplatin, doxorubicin, ifosfamide and methotrexate has been widely used to reduce the recurrence rates and improve the survival of patients with osteosarcoma. Resistance to chemotherapeutic agents is still the main problem in osteosarcoma and cancer treatment. Detection of cancer biomarkers in human serum is a novel method in diagnosis and prognosis of cancer. In clinical practice, levels of serum lactate dehydrogenase (LDH) are routinely measured in the diagnosis of myocardial infarction, liver disease and certain malignancies because it is released during tissue damage. Elevated serum LDH were found to correlate with a larger tumor burden and to predict a poor prognosis (Miao et al. 2013). Alkaline phosphatase (ALP) plays an important role in osteoarticular processes. The serum level of bone-specific ALP reflects the cellular activity of osteoblasts, and elevated serum level of ALP has been found during bone formation or increased bone turnover (Pääkkönen et al. 2013). However, there is still no effective serum indicator for diagnosis and prognosis of patients with osteosarcoma.

micro-RNA (miRNA or miR), a highly conserved non-coding RNA molecule with 18–25 nucleotides, regulates gene expression at the post-transcriptional level by binding to the 3′-untranslated regions of their target mRNAs (Ha and Kim 2014). miRNAs can control as many as 60% of protein coding genes in human (Namløs et al. 2012). miRNAs involve in multiple cellular processes, such as differentiation, proliferation, and apoptosis, which are of great importance in the development of cancer (Jones et al. 2012). With the development of biotechnologies for miRNA expression profiling, an increasing number of studies have suggested that miRNAs may be closely associated with initiation and progression of cancer. More than half of the annotated miRNAs were found to be located within fragile sites of human genome that were related to cancer (Huang et al. 2013). The level of miR-95-3p was upregulated in higher-grade glioma tissues of patients with glioma. In glioma cells, downregulation of miR-95-3p inhibits proliferation, invasion and promotes apoptosis (Fan et al. 2015). In mice, experiments showed that miR-95-3p overexpression promoted tumor growth and increased radiation resistance in tumor xenografts (Huang et al. 2013). Lawrie et al. (2008) found that high levels of miR-155a and miR-210 in serum were correlated with disease-free survival rates of patients with diffuse large B cell lymphoma. As oncogenes or tumor suppressors, circulating miRNAs may develop to be a promising diagnostic and prognostic marker for various types of cancer, including osteosarcoma, nasopharyngeal carcinoma, glioma, as well as breast, gastric, prostate and lung cancer.

There are many advantages for circulating miRNAs in serum to be promising biomarkers. First, Serum samples are more easily accessible compared with tumor tissue samples. Second, due to protection of serum, miRNAs are highly resistant to RNase-mediated degradation and other severe conditions such as extreme pH, high temperature, and repeated freeze–thaw cycles. Third, most of miRNAs are evolutionarily conserved in animal, plant and bacterial viruses. Finally, sufficient miRNAs in serum make it easy to be determined by simple and fast methods (Etheridge et al. 2011; Lodes et al. 2009; Ma et al. 2012; Park et al. 2009).

In the present study, we detected the expression levels of miR-95-3p in serum samples of osteosarcoma patients and healthy individuals. Then, the correlations between serum miR-95-3p level and clinicopathological factors or overall survival of osteosarcoma patients were evaluated. The prognostic value of miR-95-3p expression level was demonstrated for overall survival of patients with osteosarcoma.

## Methods

### Sample source

The experiments was approved by the Ethics Review Board of Zhengzhou Orthopedics Hospital. Tumor tissues and matched serum samples were collected after obtaining written informed consents from each participant.

**Fig. 1:**
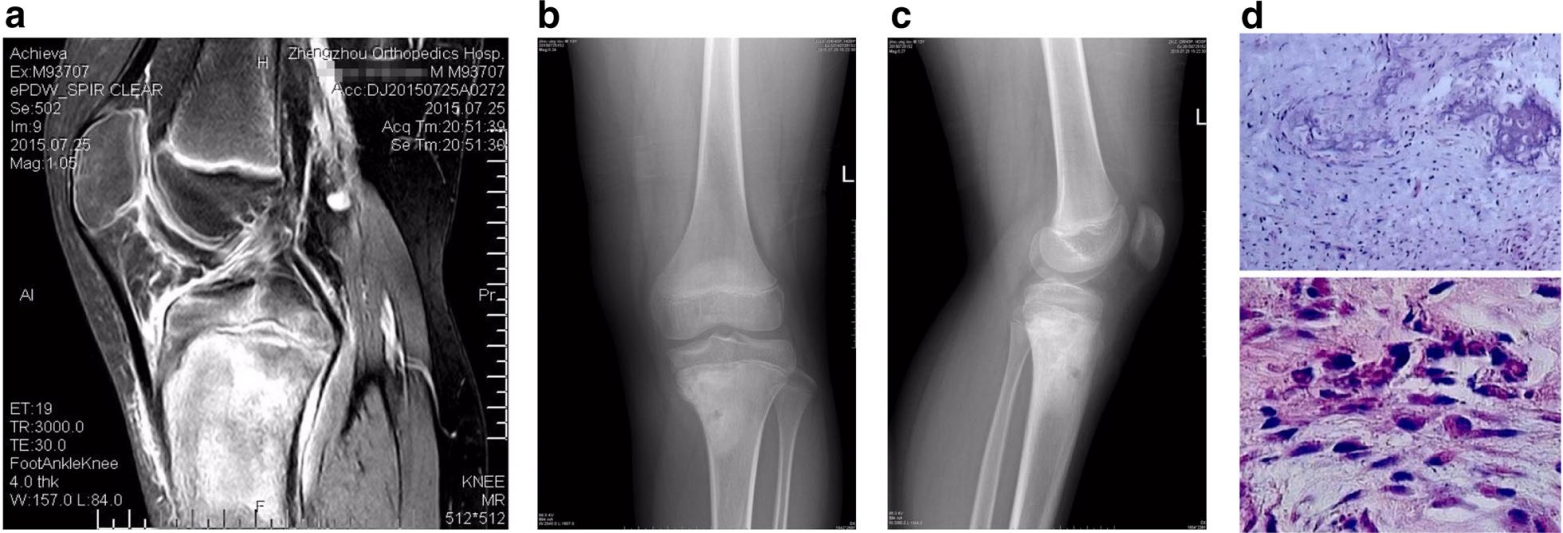
The imaging features of osteosarcoma. **a** MRI findings included osteolytic destruction, joint effusion and soft tissue mass. **b**, **c** X-ray showed osteolytic bone destruction in proximal tibia. **d**, **e** Histological data showed fusiform osteosarcoma cells

In total, 133 patients diagnosed with osteosarcoma (Fig. 1) and 133 sex- and age-matched healthy individuals from Zhengzhou Orthopedics Hospital between March 2013 and July 2015 were recruited in this study. Neither chemotherapy nor radiotherapy had been used in all of the patients before surgery treatment. Clinical information of patients was obtained from medical records and pathology reports. Tumor stage was classified according to the tumor-lymph node-metastasis classification system of the International Union against Cancer. All of the osteosarcoma patients received regular followed-up. Overall survival time was defined as the time interval from primary surgery to the date of death or last follow-up. The clinicopathological data of patients was retrospectively reviewed and summarized in Table 1.

**Table 1:** Association of serum miR-95-3p expression with clinicopathological features of osteosarcoma

Clinicopathological features	No. of cases	Expression of miR-95-3p		*P*
		Low	High	
Age (years)				
≤15	59	40	19	0.234
>15	74	57	17	
Sex				
Male	71	53	18	0.634
Female	62	44	18	
Location				
Tibia	58	44	14	0.375
Femur	31	25	6	
Splinter bone	22	16	6	
Humeral bone	15	12	3	
Elsewhere	7	5	2	
Tumor diameter (cm)				
≤5	73	52	21	0.627
>5	60	45	15	
Serum level of lactate dehydrogenase				
Elevated	72	50	22	0.325
Normal	61	47	14	
Serum level of alkaline phosphatase				
Elevated	87	64	23	0.822
Normal	46	33	13	
Clinical stage				
IIA	49	27	22	0.000
IIB/III	84	70	14	
Metastasis				
Yes	68	40	28	0.000
No	65	57	8	
Response to chemotherapy				
Good	57	32	25	0.000
Poor	76	65	11	

For each subject, 3 mL venous blood was collected from 133 patients and placed in a test tube. The whole blood samples were incubated at 37 °C for 1 h and then centrifuged immediately at 1500*g* for 15 min at 4 °C. The serum was stored in 1.5 mL RNase free tubes at −80 °C for RNA extraction.

### RNA isolation

Total RNA was isolated from fresh osteosarcoma tissue and serum samples using the miRNeasy Mini Kit (Qiagen, Valencia, CA, USA) and miRVana RNA isolation kit (Ambion Inc, Austin TX, USA) according to the manufacturer’s protocol. RNA purity and concentrations were measured with the Nanodrop 2000 (Thermo Fisher Scientific, San Jose, CA, USA). RNA integrity was detected on a agarose gel with ethidium bromide staining by electrophoresis. The RNA samples were immediately stored at −80 °C for next cDNA convertion.

### qRT-PCR

cDNA was synthesized by reverse transcription reaction using the TaqMan miRNA Reverse Transcription Kit (Applied Biosystems, Foster City, CA, USA) according to the manufacturer’s protocol. 100 ng tissue-derived RNA or 40 ng serum-derived RNA was used in the reverse transcription reactions. The expression level of precursor miR-95 was quantified using SYBR green qRT-PCR assay (Invitrogen, Carlsbad, CA, USA) and real-time PCR was performed on a MyiQ Real-Time PCR Detection System (Bio-Rad, Richmond, CA, USA). The nuclear RNA U6 was used as an endogenous control for the expression of precursor miR-95. Each sample was examined in triplicate in 20 μL volumes, including no-template controls. The following primers (Sangon Biotech, Shanghai, China) were used: precursor miR-95 forward, 5′-CTGGTGGAGGGATGGATGAA-3′; reverse, 5′-GGCCCGATCACAAACTCATC-3′; U6 forward, 5′-AACGCTTCACGAATTTGCGT-3′; reverse, 5′-CTCGCTTCGGCAGCACA-3′. The 2^−ΔΔCt^ cycle threshold method was used to qualify the relative expression level of miR-95-3p.

### Statistical analysis

Continuous variables were expressed as mean ± standard deviation (SD). Associations between serum levels of miR-95-3p and clinicopathological characteristics or survival of osteosarcoma patients were evaluated using Chi square test. Survival rate was determined using the Kaplan–Meier method and differences between groups were examined using Log-rank test. Survival data were assessed by multivariate Cox regression analysis. All *P* values were two-sided and *P* < 0.05 was considered statistically significant. The statistical analyses of all experimental data were conducted using SPSS 18.0 statistical software (SPSS Inc., Chicago, IL, USA).

## Results

### Decreased levels of serum miR-95-3p in osteosarcoma patients

**Fig. 2:**
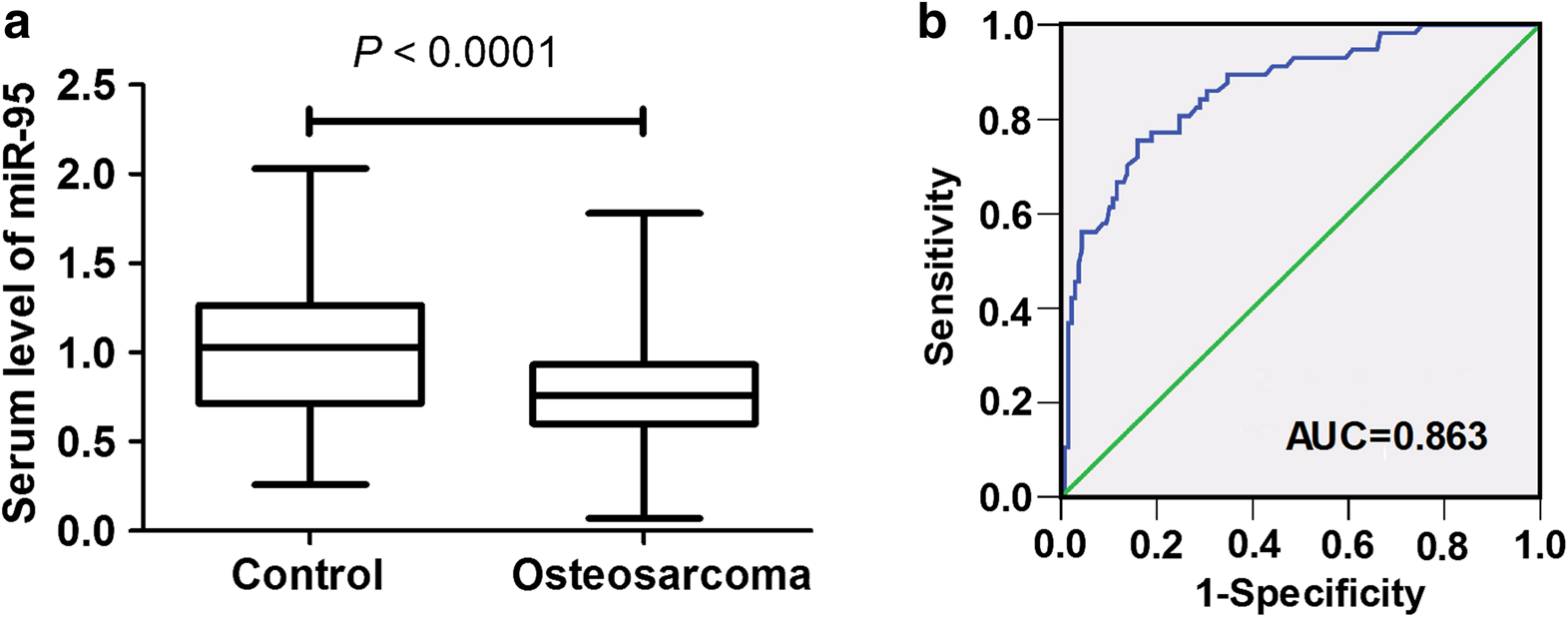
Diagnostic value of serum miR-95-3p expression for osteosarcoma patients. **a** Serum miR-95-3p expression levels in 133 osteosarcoma patients and 133 sex- and age-matched healthy controls were determined by qRT-PCR. The serum levels of miR-95-3p were significantly decreased in osteosarcoma patients in comparison with healthy controls. **b** ROC curve analysis showed that serum miR-95-3p was a novel potential biomarker for early diagnosis of osteosarcoma (AUC = 0.863)

qRT-PCR was conducted to determine the expression levels of serum miR-95-3p in patients with osteosarcoma and sex- and age-matched healthy controls. miR-95-3p expression levels were significantly decreased in serum of patients with osteosarcoma in comparison with those of healthy controls (mean ± SD 0.78 ± 0.32 vs. 1.01 ± 0.42, *P* < 0.0001, Fig. 2).

### Association between decreased serum levels of miR-95-3p expression and clinicopathologic characteristics in osteosarcoma patients

We divided the patients into two groups using the median value of serum miR-95-3p expression levels in osteosarcoma patients as a cutoff point (0.75). Of 133 osteosarcoma specimens, 69 osteosarcoma specimens (51.87%) showed low expression of miR-95-3p. To further investigate the roles of miR-95-3p in the development and progression of osteosarcoma, the association between miR-95-3p expression and clinicopathological features of patients with osteosarcoma was analyzed using Chi square test. As shown in Table 1, low-expressed miR-95-3p showed significant association with clinical stage (*P* = 0.000), metastasis (*P* = 0.000), and response to chemotherapy (*P* = 0.000). In contrast, no significant association was found between the expression level of miR-95-3p with patients’ age (*P* = 0.234), sex (*P* = 0.634), location (*P* = 0.375), and tumor diameter (*P* = 0.627). Taken together, these results demonstrated that low miR-95-3p expression in serum was associated with the progression and development of osteosarcoma.

### Decreased expression of miR-95-3p in serum associates with poor prognosis in osteosarcoma patients

**Fig. 3:**
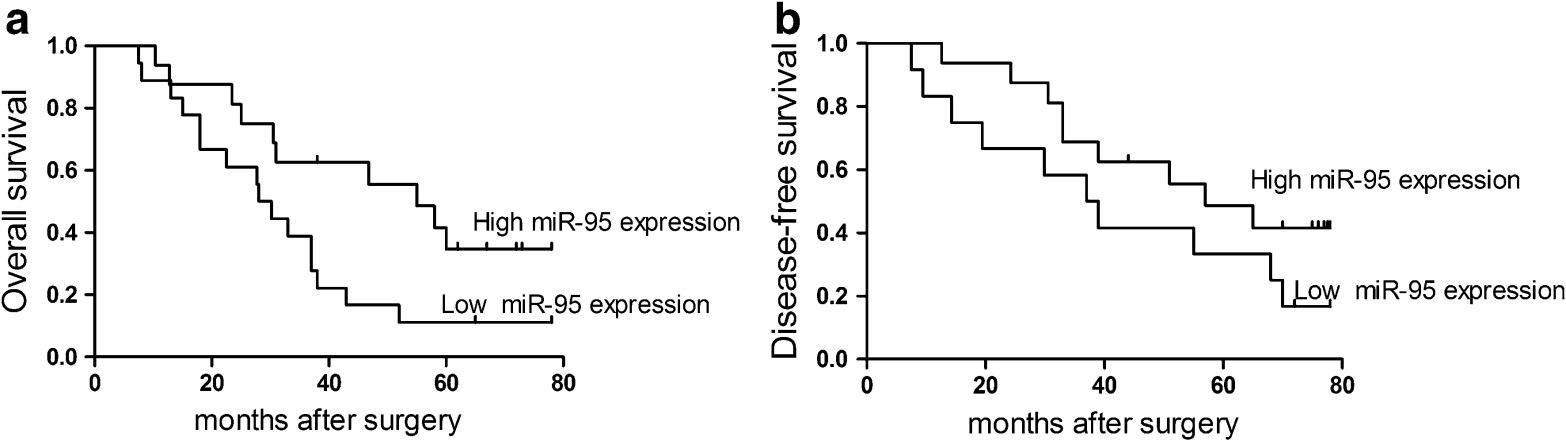
Kaplan-Meier survival curves for osteosarcoma patients with high or low expression of miR-95-3p. The overall survival curves and disease-free survival curves for two groups of osteosarcoma patients with low and high expression of serum miR-95-3p. **a** The overall survival rate of osteosarcoma patients with high miR-95-3p expression were significantly higher than those with low miR-95-3p expression (*P* = 0.017). **b** The disease-free survival rate of osteosarcoma patients with high miR-95-3p expression were significantly higher than those with low miR-95-3p expression (*P* = 0.008)

The correlation between miR-95-3p expression level, overall survival (Fig. 3a, *P* = 0.017) and disease-free survival (Fig. 3b, *P* = 0.008) of the patients with osteosarcoma was assessed using Kaplan–Meier survival analysis. The Kaplan–Meier curves for overall survival showed that osteosarcoma patients with high miR-95-3p expression in serum survived significantly longer than those with low miR-95-3p expression (log-rank test, *P* = 0.017, shown in Fig. 3a). Multivariate Cox proportional hazards model analysis suggested that expression level of serum miR-95-3p was a significant independent prognostic factor of overall survival for patients with osteosarcoma (HR 4.22, 95% CI 2.314–8.072, *P* = 0.014, shown in Table 2).

**Table 2:** Multivariate survival analysis of overall survival in 133 osteosarcoma patients

Variables	Hazard ratio	95% CI	*P*
Sex	1.16	0.674–1.818	0.557
Age	0.87	0.469–2.473	0.682
Location	0.94	0.693–2.197	0.728
Tumor diameter	2.74	1.825–5.426	0.066
Clinical stage	3.12	1.725–6.429	0.018
Metastasis	3.35	1.794–7.531	0.031
miR-95-3p expression	4.22	2.314–8.072	0.014

## Discussion

miRNAs play crucial roles in multiple cellular processes and are associated with development and progression of various cancers and diseases. Several circulating miRNAs were identified as promising biomarkers and provided guidance for early detection and blood screening for occult malignancy, including glioma (Wang et al. 2012), breast (Ng et al. 2013, Schrauder et al. 2012), lung (Yan et al. 2015), prostate (Sita-Lumsden et al. 2013), and colorectal cancer (Menéndez et al. 2013). The finding that serum miRNAs act as potential biomarkers for cancer overcomes the disadvantages of collecting tissue samples from patients through biopsy or surgery. Recently, A growing body of studies have showed that aberrant expression of miRNAs is associated with prognosis and progression in human osteosarcoma (Azam et al. 2015; Kobayashi et al. 2014; Ouyang et al. 2013).

miR-95-3p were downregulated in both high and low grade osteosarcoma tissues compared with normal controls (Novello et al. 2013). Hwang et al. (2015) revealed that overexpression of microRNA-95-3p inhibited brain metastasis of lung adenocarcinoma through downregulation of cyclin D1. Their data suggested that targeting miR-95-3p may be a novel therapeutic strategy for brain metastasis of lung adenocarcinoma cells. However, the potential roles of miR-95-3p in pathogenesis and prognosis of osteosarcoma are not yet illustrated. In this study, the expression levels of miR-95-3p in serum were determined in osteosarcoma patients and healthy controls. The diagnostic value of serum miR-95-3p expression for osteosarcoma patients was assessed. Our data showed that miR-95-3p could distinguish osteosarcoma patients from healthy controls efficiently (AUC = 0.863). The AUC value is an indicator of efficacy of the assessment system. In addition, we found that downregulation of serum miR-95-3p in osteosarcoma patients was significantly associated with unfavorable clinicopathological characteristics, including metastasis, clinical stage and chemotherapy resistance. These findings suggested that a lower expression level of serum miR-95-3p was implicated in the pathogenesis and progression of osteosarcoma. Furthermore, Kaplan–Meier analysis and log-rank test for overall survival showed that patients with low serum level of miR-95-3p had remarkably shorter overall survival time than those with high expression of serum miR-95-3p. The multivariate Cox proportional hazards model suggested that clinical stage, metastasis, and decreased expression of serum miR-95-3p were detected to be as independent prognostic factors for overall survival.

We firstly demonstrated that decreased serum level of miR-95-3p may act as a diagnostic and prognostic biomarker for patients with osteosarcoma. Nevertheless, miRNA expression is regulated by genetic and epigenetic factors in human and there are some possible causes for the dysregulation of miRNA in various malignancy. For example, MYC and MYCN genes regulate the expression of miR-9 in breast cancer, and DNA methylation influences miR-9 expression in colorectal cancer (Vinci et al. 2013). The expression of miR-95-3p was elevated in colorectal carcinoma, Hela cells and pancreatic carcinoma, which suggested that miR-95-3p may play an oncogenic role in these carcinomas (Huang et al. 2011). In the present study, we demonstrated that miR-95-3p worked as an anti-oncogenic miRNA in osteosarcoma and decreased expression of miR-95-3p was a statistically significant risk factor reducing overall survival in osteosarcoma patients. However, the molecular mechanism of miR-95-3p in osteosarcoma tumorigenesis and progression has not yet been elucidated.

## Conclusion

Our results suggested that miR-95-3p expression may decreased in serum of osteosarcoma patients. Detection of serum miR-95-3p levels may have clinical potentials as a valuable diagnostic and prognostic biomarker for osteosarcoma patients. Further studies should be conducted to clarify the exact mechanism of miR-95-3p in osteosarcoma.
